# Therapeutic bronchoscopy followed by sequential radiochemotherapy in the management of life-threatening tracheal adenoid cystic carcinoma: a case report

**DOI:** 10.1186/s13256-022-03452-1

**Published:** 2022-06-20

**Authors:** Mia Elhidsi, Jamal Zaini, Aziza Ghanie, Aida Lutfi Huswatun, Romi Beginta, Susan Hendriarini Mety, Elisna Syahruddin

**Affiliations:** 1 Department of Pulmonology and Respiratory Medicine, Faculty of Medicine, Universitas Indonesia, Persahabatan National Respiratory Referral Hospital, Jakarta, Indonesia; 2 Indonesian Bronchoscopy Society, Perbronki, Jakarta, Indonesia; 3 Faculty of Medicine, Universitas UPN Veteran Jakarta, Jakarta, Indonesia; 4 Department of Radiology, Persahabatan National Respiratory Referral Hospital, Jakarta, Indonesia; 5 Department of Radiotherapy, Persahabatan National Respiratory Referral Hospital, Jakarta, Indonesia; 6 Department of Pathology, Persahabatan National Respiratory Referral Hospital, Jakarta, Indonesia; 7 Department of Thoracic, Cardiac, and Vascular Surgery, Persahabatan National Respiratory Referral Hospital, Jakarta, Indonesia; 8 Thoracic, Cardiac, and Vascular Surgery Division, Department of Surgery, Faculty of Medicine, Universitas Indonesia, Jakarta, Indonesia

**Keywords:** Therapeutic bronchoscopy, Adenoid cystic carcinoma, Radiochemotherapy, Central airway obstruction, Case report

## Abstract

**Background:**

Adenoid cystic carcinoma of the lung is a distinctive salivary-gland-type malignant epithelial neoplasm that rarely presents as a primary tumor of the respiratory tract. Complete surgical resection remains the treatment of choice for adenoid cystic carcinoma. We present a case of large ACC tumors that caused severe central airway obstruction and were effectively treated with therapeutic bronchoscopy followed by radiotherapy and chemotherapy.

**Case presentation:**

A 31-year-old Malay Indonesian female patient who was a nonsmoker and had no family history of cancer was admitted to the emergency ward because of worsening breathlessness accompanied by stridor since 1 week prior. Chest computed tomography revealed segmental atelectasis of the left lung; a mass on the left main bronchus, with infiltrates in segments 1, 2, and 3 of the left lung; and consolidation in the left inferior lobe, with narrowing of the main left bronchus. Lobulated masses obstructing almost the entire distal trachea up to the carina and the entire left main bronchus were found on bronchoscopy. Owing to the large tumors causing severe central airway obstruction, the medical team decided to perform central airway mass removal through rigid bronchoscopy. A neodymium-doped yttrium-aluminum-garnet laser was used first to facilitate mass shrinkage. After the laser treatment, mechanical mass removal using a rigid scope was performed. The tracheal and carinal lumens were opened to > 50% of their diameter, with the left main bronchus lumen opened only slightly. After the treatment, the patient was stable, and no stridor was found. Adjuvant intensity-modulated radiotherapy and chemotherapy were performed after the therapeutic bronchoscopy. At the end of the entire treatment, reevaluation by thoracic computed tomography scan and bronchoscopy revealed no remaining mass.

**Conclusions:**

In cases of nonresectable large adenoid cystic carcinoma tumors with life-threatening central airway obstruction, therapeutic bronchoscopy followed by sequential radiochemotherapy might achieve a complete response outcome.

## Background

Adenoid cystic carcinoma (ACC) of the lung is a distinctive salivary-gland-type malignant epithelial neoplasm that rarely presents as a primary tumor of the respiratory tract. It accounts for 10% of all salivary gland tumors, 1–2% of all head and neck malignancies, and < 1% of all bronchopulmonary cancers [1, 2]. However, its incidence was reported to be higher in tracheal tumors, at 15% [3]. Owing to its relatively low incidence, its clinical presentation and disease course have not yet been fully elucidated.

According to current evidence, complete surgical resection is still the treatment of choice for patients with ACC [4]. Despite the effectiveness of complete surgical resection, cases that require emergency treatment or those with large tumors involving the trachea and lungs that have ill-defined margins pose a significant challenge to surgery. Therefore, we present a case of large ACC tumors causing a life-threatening central airway obstruction that showed complete response to nonsurgical management with laser and rigid bronchoscopic debulking followed by sequential radiochemotherapy.

## Case presentation

A 31-year-old Malay Indonesian female patient who was a nonsmoker and had no family history of cancer was admitted to the emergency ward with worsening breathlessness accompanied by wheezing since 1 week prior. The patient also had recurring hemoptysis, which started 2 years before and worsened in the past 6 months. Previously, she received antituberculosis treatment for 6 months, along with inhaled long-acting bronchodilator and steroid therapies. However, no clinical improvement was observed.

**Fig. 1:**
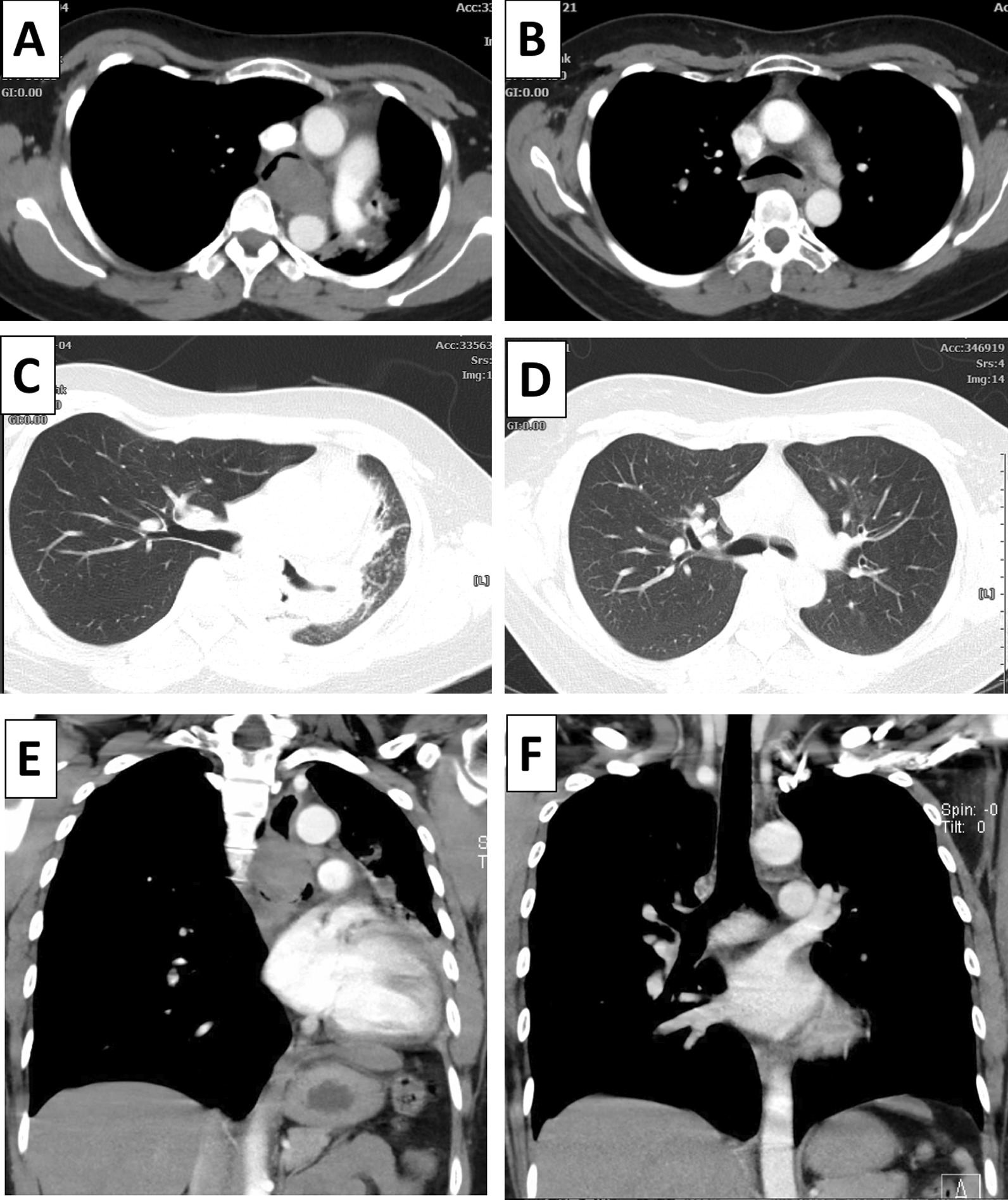
Thoracic computed tomography (CT) scan before and after treatment. **A** Axial mediastinal window of the chest CT scan taken before the procedure, showing a mass on the left main bronchus. **B** Axial mediastinal window of the chest CT scan taken 2 months after radiotherapy and chemotherapy, showing no visible mass. **C** Axial lung window of the CT scan taken before the procedure, showing segmental atelectasis of the left lung. **D** Axial lung window of the chest CT scan taken 2 months after radiotherapy and chemotherapy, showing no pulmonary mass. **E** Coronal mediastinal window of the chest CT scan taken before the procedure, showing a mass on the left main bronchus. **F** Coronal mediastinal window of the chest CT scan taken 2 months after radiotherapy and chemotherapy, showing no pulmonary mass

Upon physical examination, severe dyspnea with stridor and decreasing vesicular breath sounds in the left lung were found. Chest radiography and computed tomography (CT) performed 1 week earlier revealed a mass on the left main bronchus (Fig. 1A, E) and segmental atelectasis of the left lung (Fig. 1C), with infiltrates in segments 1, 2, and 3 of the left lung, along with consolidation in the left inferior lobe and narrowing of the main left bronchus.

**Fig. 2:**
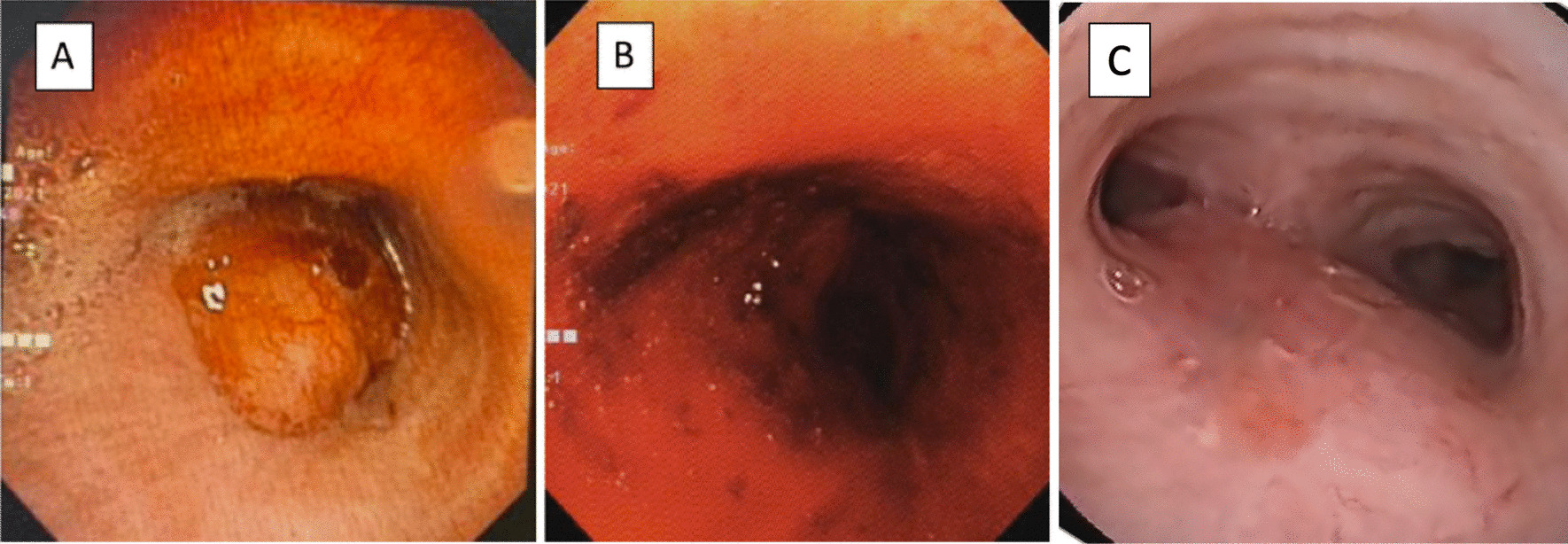
Bronchoscopic view of the tracheal adenoid cystic carcinoma before and after bronchoscopic mass removal. **A** Bronchoscopic view before the bronchoscopic mass removal, showing lobulated masses obstructing almost the entire distal tracheal lumen. **B** Bronchoscopic view after the bronchoscopic mass removal, showing the tracheal lumen opened to >50% of its diameter. **C** Bronchoscopic view 2 months after radiotherapy and chemotherapy, showing no endoluminal mass and only slight malacia

The multidisciplinary medical team decided that the case was inoperable due to the large tumors, severe central airway obstruction, and large lung involvement; thus, therapeutic bronchoscopy was the best feasible treatment plan for the patient. After discussing with the patient and her family, 3 days after the patient’s admission, the medical team decided to perform bronchoscopic examination and tracheobronchial mass removal through rigid bronchoscopy. Lobulated masses obstructing almost the entire distal tracheal lumen up to the carina and the entire left main bronchus lumen were found on bronchoscopy (Fig. 2A). A neodymium-doped yttrium-aluminum-garnet laser was used first to facilitate mass shrinkage. After the laser treatment, mechanical debulking using a rigid scope was performed. Intraprocedure bleeding was managed with argon plasma coagulation (APC) through posttherapeutic bronchoscopy. The tracheal and carinal lumens were opened to > 50% of their diameter, with the left main bronchus lumen opened only slightly (Fig. 2B). After bronchoscopic mass removal, the patient was stable, and no stridor was found. The dyspnea was relieved with 96–98% peripheral oxygen saturation in room air.

**Fig. 3:**
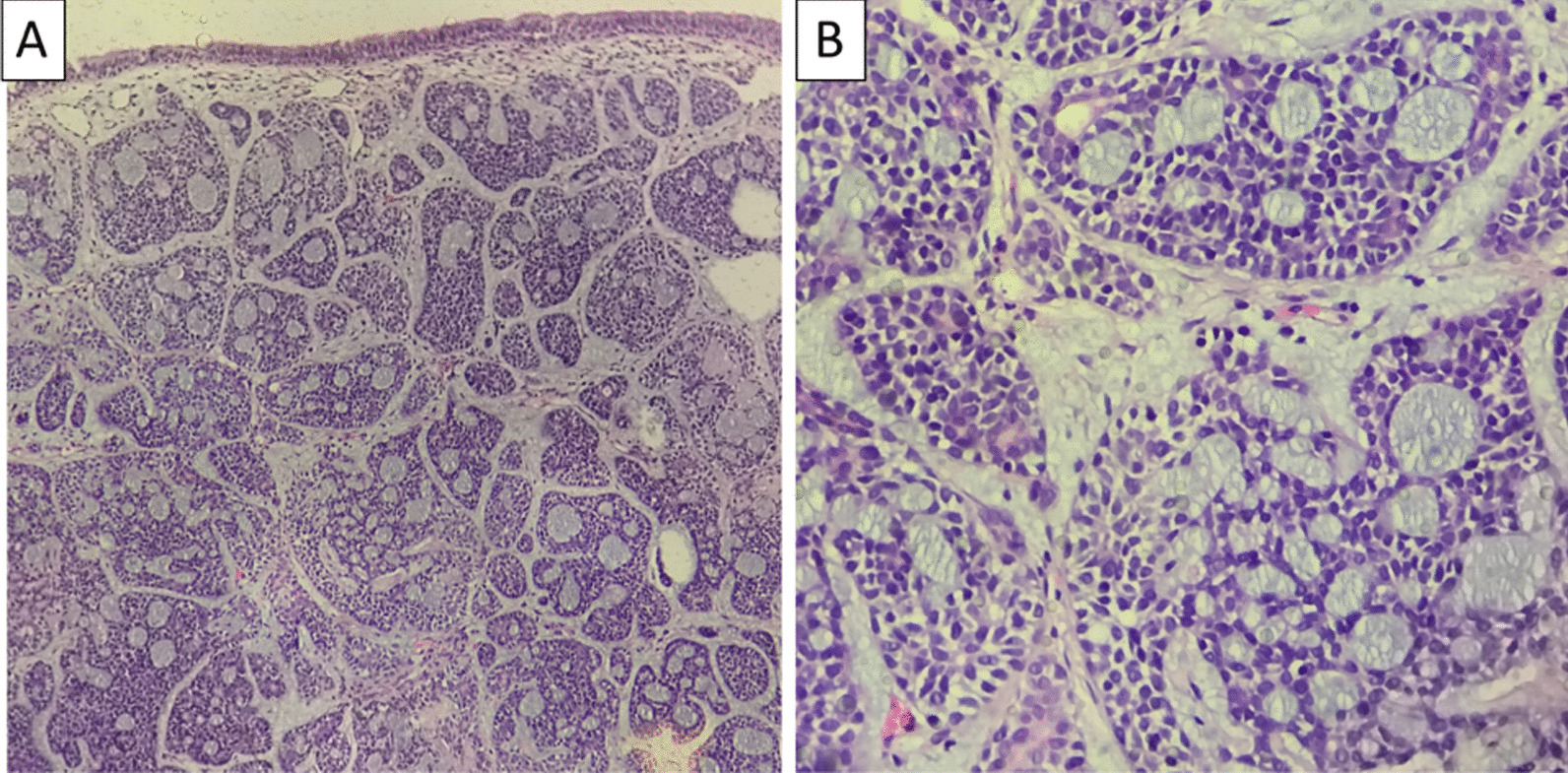
Histopathological examination result showing a tumor mass with cribriform structure and solid islands. **A** Hematoxylin–eosin staining at 40× magnification. **B** Hematoxylin–eosin staining at 200×

Pathological examination using hematoxylin–eosin staining confirmed the presence of a tumor mass with solid islets and a cribriform structure. The tumor cells had relatively uniform and basaloid nuclei. Mitosis was rarely found. The bronchial mucosa was visible above the surface of the tumor, and no perineural invasion was found (Fig. 3A, B). The pathological diagnosis was therefore concluded to be grade 2 adenoid cystic carcinoma. Molecular or cancer-related genetic testing and immunohistochemical staining to confirm the diagnosis were not performed because of insurance limitations.

Definitive intensity-modulated radiotherapy (IMRT) targeting the tracheobronchial mass, including the distal trachea, carina, and left main bronchus as gross tumor volume (GTV), was commenced 2 weeks after the therapeutic bronchoscopy. The clinical target volume (CTV) was GTV + 1 cm, and the planning target volume was CTV + 0.5 cm. The total dose plan amounted to 60 Gy, but the radiation dose could be delivered at 40 Gy and administered in 20 fractions of 2 Gy. After 20 fractions of radiation over 4 weeks, the patient developed esophagitis and dysphagia. Positioning the patient with thermoplastic masks was difficult because of mucous hypersecretion and persistent coughing. Two weeks after the radiotherapy series, the patient received a chemotherapy regimen consisting of carboplatin with an area under the curve of 5 and paclitaxel 175 mg/m^2^, given three times weekly for a total of six cycles. The patient tolerated the treatment well and did not experience any toxicities.

**Fig. 4:**
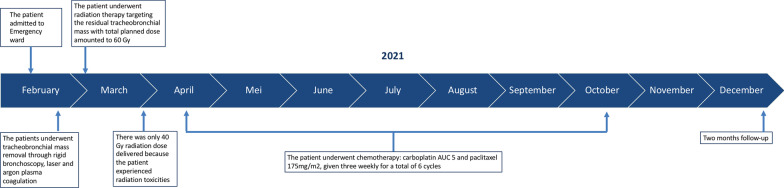
Representative timelines of the patient treatment history

Two months after completion of the treatment, the patient had no dyspnea. In reevaluation by chest CT scan, no mass in either lung or the mediastinum and no lymph node enlargement were visible (Fig. 1D). The trachea and main bronchi were normal (Fig. 1B, F). Bronchoscopy also revealed no mass and only slight malacia (Fig. 3C). Finally, the patient was planned to undergo routine evaluation by thoracic CT scan every 3–6 months. Figure 4 shows the patient’s treatment and follow-up timelines.

## Discussion

Malignant endoluminal central airway obstruction still proves challenging for clinicians and thus requires individualized strategies with specific interventions that can be performed in a particular healthcare center. In the present case, surgery was not performed because of the tumor location and size. Previous studies have reported that surgical management of ACC tracheobronchial cases was only indicated for locoregional tumors. Complete tumor resection and negative airway margins were considered indicators of successful surgery [5]. On the other hand, nonsurgical interventions may include bronchoscopic therapy, external-beam radiotherapy, and chemotherapy [6].

In life-threatening cases, immediate-effect bronchoscopy is the modality of choice. We chose a combination of rigid scope, laser, and argon plasma coagulation in the present case. Lasers are capable of resecting and vaporizing tissues and therefore produce an excellent coagulation effect [7, 8]. The combination of laser treatment with rigid and flexible bronchoscopy has shown good performance in intraluminal mass removal [9, 10]. Rigid bronchoscopy by skilled bronchoscopists is superior to its flexible counterparts because of its large caliber and rigid tube that allow operators to access and control the airway, providing ventilation, while accommodating the laser, APC instruments, and suction simultaneously [11]. The scope barrel neatly functions as a tampon in cases of hemorrhage [12]. In the present case, tumor shrinkage by laser followed by rigid bronchoscopy debulking was proven to be safe, with no massive bleeding or airway laceration. This finding is in line with the excellent efficacy and safety of the rigid scope in airway recanalization reported in a previous study [13]. Post-laser-treatment APC was also used in our case because it has been reported to achieve good hemostasis for mucosal bleeding and to confer a minimal risk of airway perforation [14].

ACC has been reported to exhibit low radiation sensitivity, but some studies have reported good radiotherapy response [15, 16]. After the therapeutic bronchoscopy, IMRT was planned at a dose of 60 Gy to avoid the risk of complications associated with higher doses [5]. As patients in previous reports experienced esophagitis due to radiation toxicity, only 40 Gy was delivered to our patient. In this case, chemotherapy was administered after the radiotherapy. Current evidence shows that radiochemotherapy after therapeutic bronchoscopy has a good clinical outcome [17]. Several case studies have also reported that combining carboplatin and paclitaxel chemotherapy regimens with radiotherapy could be an effective option for unresectable ACC [18, 19]. For our case, follow-up examinations with serial CT scans over a posttreatment course of 5 years were planned.

## Conclusion

Surgical resection is a widely recommended treatment for localized tracheal ACC. In nonresectable large ACC tumors with life-threatening central airway obstruction, therapeutic bronchoscopy may be lifesaving, and sequential radiochemotherapy after mass removal might achieve a complete response outcome.

## Data Availability

The datasets used in the study are available from the corresponding author on reasonable request.

## Abbreviations

ACC: Adenoid cystic carcinoma
CT: Computed tomography
APC: Argon plasma coagulation
IMRT: Intensity-modulated radiotherapy
